# Characterizing early adopters of the rural emergency hospital designation

**DOI:** 10.1093/haschl/qxag056

**Published:** 2026-03-11

**Authors:** Jonah M Graves, R J Waken, Fengxian Wang, Paula Chatterjee, Karen E Joynt Maddox

**Affiliations:** Pulmonary and Critical Care Medicine Division, Department of Medicine, Washington University School of Medicine, St Louis, MO 63110, United States; Center for Advancing Health Services, Policy and Economics Research, Washington University School of Medicine, St Louis, MO 63110, United States; Center for Advancing Health Services, Policy and Economics Research, Washington University School of Medicine, St Louis, MO 63110, United States; Division of Biostatistics, Institute for Informatics, Data Science, and Biostatistics, Washington University School of Medicine, St Louis, MO 63110, United States; Center for Advancing Health Services, Policy and Economics Research, Washington University School of Medicine, St Louis, MO 63110, United States; Cardiovascular Division, Department of Medicine, Washington University School of Medicine, St Louis, MO 63110, United States; Department of Medicine, Perelman School of Medicine, University of Pennsylvania, Philadelphia, PA 19104, United States; The Parity Center, Perelman School of Medicine, University of Pennsylvania, Philadelphia, PA 19104, United States; Center for Advancing Health Services, Policy and Economics Research, Washington University School of Medicine, St Louis, MO 63110, United States; Cardiovascular Division, Department of Medicine, Washington University School of Medicine, St Louis, MO 63110, United States

**Keywords:** rural health, health services accessibility, hospital closure

## Introduction

Over the last 2 decades, rural communities across the United States have lost access to inpatient and emergency health care services due to hospital closures. Between 2005 and 2025, 195 rural hospitals either converted to outpatient facilities or closed entirely, and currently more than 700 rural hospitals are at risk of closure.^1,2^ In an effort to maintain access to emergency services in underserved and isolated areas, the Consolidated Appropriations Act of 2021 created a Rural Emergency Hospital (REH) designation, allowing small rural and critical access hospitals (CAHs) the option of converting to stand-alone emergency departments capable of providing up to 24 hours of observation care (but forgoing their inpatient capability) in exchange for enhanced federal funding. The program took effect on January 1, 2023.

Previous work identified that more than 1500 hospitals may be eligible to receive REH designation,^3^ with 681 hospitals identified as most likely to convert to REH status due to low emergency department volumes.^4^ However, little is known about the early adopters of the REH designation in the first 2 years of the program. We aimed to characterize these REH early adopters to provide insight on the motivations and circumstances influencing program participation, the potential scale of future program adoption, and its potential impact on access to care in rural areas.

## Methods

We performed a descriptive cross-sectional analysis of preconversion hospital characteristics of participating REHs as of December 2025 and nonconverted hospitals eligible for REH designation (REH-eligible) within the same Hospital Referral Regions (HRRs). Hospital data were primarily sourced from the American Hospital Association 2022 Annual Survey and 2022 Medicare Cost Reports. A detailed description of data sources and methods, along with variable-level missingness, is available in the eMethods. We compared characteristics between REH and REH-eligible facilities using the independent-samples Wilcoxon test for continuous variables and Fisher's exact test for categorical variables. Prior to comparison, financial characteristics were winsorized at the 5th and 95th percentiles. To account for multiple comparisons, we considered *P* < .002 (0.05/26 comparisons) significant. Analyses were performed in R version 4.4.3.

## Results

As of December 2025, 42 hospitals received REH designation, located in 30 HRRs across 18 states (eFigure). There were 447 REH-eligible facilities, in HRRs with at least 1 REH, included for comparison.

In 2022, 21 (50%) REHs were CAHs, 12 (28.6%) belonged to a health system, and none had a medical school affiliation. The majority of REHs were government-owned (54.8%) or nonprofit (33.3%), and REHs had a median (range) of 22 (2–48) hospital beds (Table 1). The REH and REH-eligible facilities were similar with regard to their ownership, size, medical school and health system affiliations, and star ratings.

**Table 1:** Preconversion characteristics of REH and REH-eligible hospitals.

	REH (*n* = 42)	REH-eligible (*n* = 447)	*P* value
**General characteristics**			
Critical access hospital, *n* (%)	21 (50.0)	318 (71.1)	.008
Ownership, *n* (%)			.31
Government	23 (54.8)	196 (43.8)	
Nonprofit	14 (33.3)	203 (45.4)	
Private	5 (11.9)	48 (10.7)	
Medical school affiliation, *n* (%)	0 (0.0)	19 (4.3)	.39
Health system membership, *n* (%)	12 (28.6%)	215 (48.1%)	.02
No. of hospital beds, median (range)	22 (2-48)	25 (6-49)	.08
CMS star rating,^a^ median (range)	2 (2-4)	3 (1-5)	.02
**Utilization characteristics, median (range)**			
ED visits	3021.0 (0.0-23002.0)	4319.0 (0.0-34619.0)	.09
Observation days	99.5 (0.0-883.0)	245.0 (0.0-3627.0)	.003
Inpatient days	405.0 (0.0-2153.0)	912.0 (0.0-9276.0)	<.001
ICU days	0.0 (0.0-1101.0)	0.0 (0.0-2278.0)	.05
Case mix index	1.2 (1.0-1.5)	1.4 (0.9-2.4)	<.001
**Staffing characteristics**			
Physician privileges, median (range)	38.5 (3.0-105.0)	70.0 (1.0-466.0)	.02
Hospitalists provide care, *n* (%)	7 (16.7)	133 (29.8)	.09
Full time RN, median (range), %	14.0 (3.0-92.0)	25.0 (2.0-190.0)	<.001
Full time RT, median (range), %	1.0 (0.0-7.0)	2.0 (0.0-18.0)	.01
Full time pharmacist, median (range), %	0.0 (0.0-4.0)	1.0 (0.0-14.0)	.002
**Service availability, *n* (%)**			
Neurological services	4 (9.5)	83 (18.6)	.25
Oncology services	1 (2.4)	123 (27.5)	<.001
Orthopedic services	6 (14.3)	189 (42.3)	<.001
Cardiology services	6 (14.3)	149 (33.3)	.01
Outpatient surgery	11 (26.2)	248 (55.5)	<.001
Hemodialysis	1 (2.4)	65 (14.5)	.04
**Financial characteristics,** ^b^ **mean (SD)**			
Total margin, %	−6.4 (14.8)	1.0 (11.1)	<.001
Operating margin, %	−33.6 (24.7)	−15.8 (20.4)	<.001
Days cash on hand	63.3 (85.8)	71.0 (82.1)	.35
Current ratio^c^	2.9 (3.6)	3.4 (3.3)	.06
Uncompensated care,^d^ %	5.6 (4.4)	4.3 (3.6)	.07

Abbreviations: CMS, Centers for Medicare and Medicaid Services; ED, emergency department; ICU, intensive care unit; REH, Rural Emergency Hospital; RN, registered nurse; RT, respiratory therapist.

^a^CMS star rating, on a scale of 1 to 5 (5 being the best), is assigned based on quality metrics relating to mortality, safety of care, readmission, patient experience, and timely and effective care.

^b^Financial characteristics are winsorized at the 5th and 95th percentiles.

^c^The current ratio represents the ratio of current assets to current liabilities.

^d^Uncompensated care is represented as the proportion of the total cost of uncompensated care to the total operating expenses.

Compared with REH-eligible facilities, REHs had significantly fewer inpatient days (median of 405 vs 912 days; *P* < .001) and lower case mix indices (median of 1.2 vs 1.4; *P* < .001). Significantly fewer REHs offered oncology services, orthopedic services, and outpatient surgery (*P* < .001), and there was a trend toward fewer staffing resources among REHs, including significantly fewer full-time registered nurses at REHs (median of 14 vs 25; *P* < .001). In 2022, REHs had lower total margins (−6.4% vs 1.0%; *P* < .001) and lower operating margins (−33.6% vs −15.8%; *P* < .001) relative to REH-eligible hospitals and trended toward fewer days of cash on hand, lower current ratios, and higher proportions of uncompensated care.

## Discussion

In 2022, the year prior to REH program implementation, the 42 early adopters of the REH designation were significantly less resourced than nonparticipating eligible hospitals in the same HRRs, offering fewer hospital services with lower inpatient volumes and worse financial profiles. Overall, we found that adoption of REH designation was relatively low in the first 2 years following policy implementation, with less than 3% of the 1500 plausibly eligible hospitals joining the program. By comparison, in the 2 years following implementation of the CAH designation, more than 100 hospitals received CAH designation.^5^ Whether this is the result of transiently abnormal health care utilization and financial resources during the COVID-19 pandemic or is a sign of low interest among rural hospitals and communities remains to be seen.

We also found that hospitals that converted to REH status were structurally similar, in terms of ownership and affiliations, but markedly lower-volume and more financially strained than non-converters. This may suggest that, ultimately, the program may only be enticing to the most financially underresourced hospitals that are otherwise facing closure, and may offer an acceptable off-ramp for those facilities.

An important limitation of our study is the lack of available quality data for most hospitals. The majority of REHs and REH-eligible hospitals were CAHs in 2022 and thus exempt from mandated public reporting of quality metrics to the Centers for Medicare and Medicaid Services (CMS). While the available CMS star ratings showed a similar distribution between REH and REH-eligible hospitals, a rating was only available for a minority of facilities. Thus, whether quality metrics associate with REH conversion remains uncertain.

Ultimately, the impact that REH designation has on access to emergency and inpatient services, quality of care, and outcomes will depend on its implementation in the coming years. Further work is warranted to monitor the ongoing adoption of the REH designation and to evaluate both its efficacy at maintaining access to care in vulnerable areas and its impact on health outcomes.

## Supplementary Material

qxag056_Supplementary_Data
