# Supplementary material for: Characterizing early adopters of the rural emergency hospital designation
Source: Health Aff Sch. 2026 Mar 11;4(3):qxag056. doi: 10.1093/haschl/qxag056 (PMC13032875; doi:10.1093/haschl/qxag056)
Supplement: qxag056_Supplementary_Data [file qxag056_supplementary_data.zip › REH_supplement_revision_clean.docx]

**Characterizing early adopters of the Rural Emergency Hospital designation**

Jonah M. Graves, MD, RJ Waken, PhD, Fengxian Wang, PhD, Paula Chatterjee, MD MPH, Karen E. Joynt Maddox, MD MPH

**Supplement**

**Table of Contents:**

**eFigure.** *Page 2*

**eMethods.** *Page 3*

**eTable.** *Page 4*

**eReferences.** *Page 6*

**
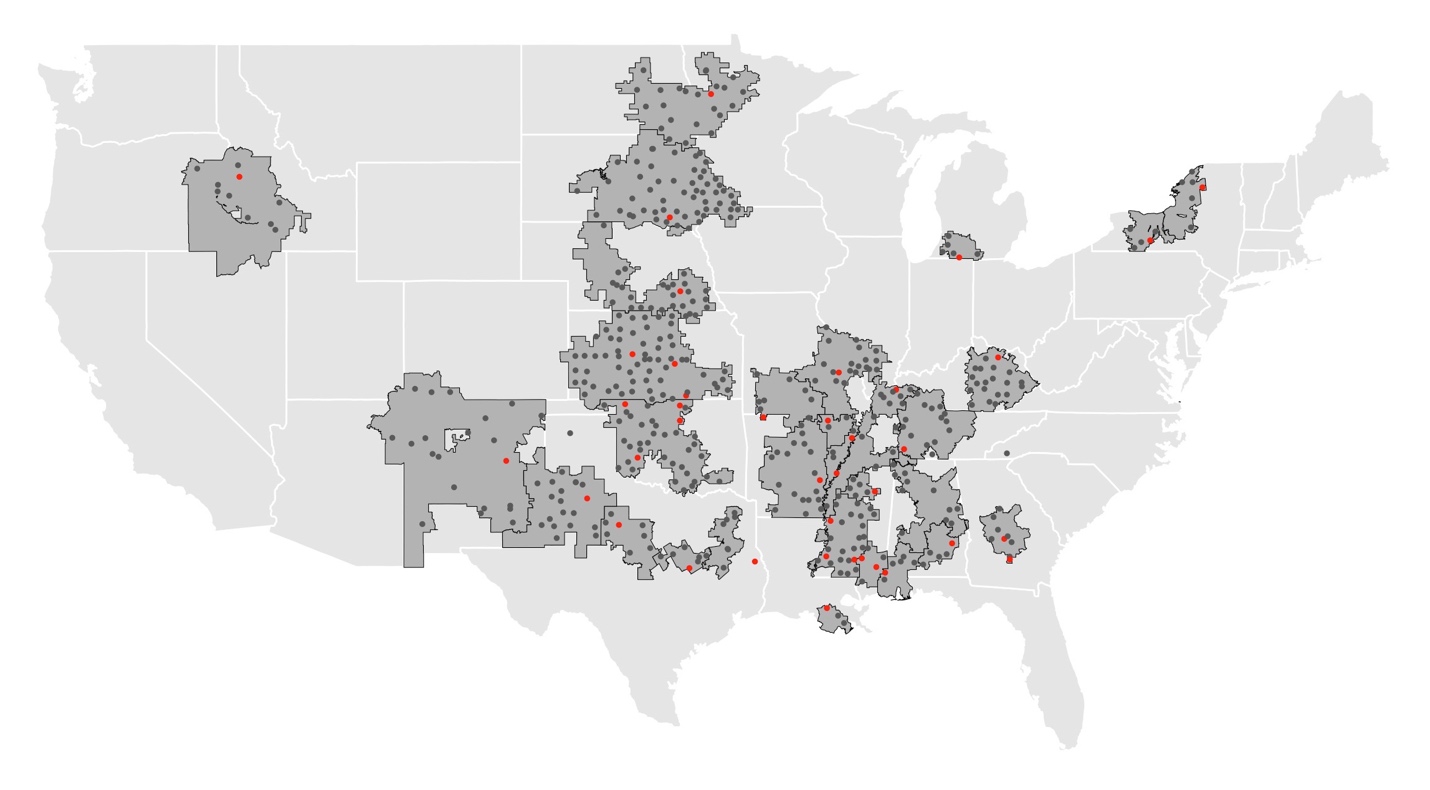
**

**eFigure: Locations of Rural Emergency Hospitals (REH) and eligible but non-participating facilities in the same Hospital Referral Regions (HRR).** As of December 2025, 42 facilities converted to REH status (red dots) among 30 HRRs across 18 states. There were 447 hospitals eligible for future REH designation in the United States in the same HRRs (dark grey dots). HRRs are outlined in black and shaded in dark gray.

**eMethods**

Data Processing:

First, the 2022 Center for Medicare and Medicaid Services (CMS) Cost Report file (CMS-2552-10) was ascertained and relevant variables were extracted.^1,2^ Preprocessed financial characteristic data from the 2022 Medicare Cost Reports relevant hospital characteristics from the 2022 American Hospital Association (AHA) Annual Survey data were ascertained from Wharton Research Data Services and linked via hospital CMS Certification Number (CCN).^3^ CMS star ratings were ascertained from CMS archived data and were linked via CCN.^4^

Facilities that had converted to Rural Emergency Hospital (REH) status as of December 2025 were identified using the December 2025 CMS Hospital Enrollment File.^5^ Any remaining hospitals that were eligible for conversion to REH status (REH-eligible, defined as critical access hospitals *or* rural hospitals with ≤50 acute care beds) were identified and labeled. For the purposes of identifying eligible facilities, rural was defined based on hospitals’ self-reported standard geographic classification on the Medicare Cost Report (Form CMS-2552-10, Worksheet S-2 Part I, line 22.03, column 1).

A detailed list of variables and their sources along with missingness is available in **eTable.**

| **eTable**   \|  \| **Missingness, n (%)** \| \|  \| \| --- \| --- \| --- \| --- \| \|  \| **REH**  **(N=42)** \| **REH Eligible**  **(N=447)** \| **Source (Code)^a^** \| \|  \| \| **General characteristics** \|  \|  \|  \| \| **Critical access hospital** \| 0 (0.0) \| 0 (0.0) \| Cost Reports (ALPHA, S200001, 10500, 100) \| \| **Ownership** \| 0 (0.0) \| 0 (0.0) \| Cost Reports (ALPHA, S200001, 2100, 100) \| \| **Medical school affiliation** \| 3 (7.1) \| 7 (1.6) \| AHA (MAPP5) \| \| **Health system membership^b^** \| 30 (71.4) \| 232 (51.9) \| AHA (MHSMEMB) \| \| **Hospital beds** \| 1 (2.4) \| 2 (0.4) \| Cost Reports (NMRC, S300001, 100, 200) \| \| **CMS hospital rating** \| 33 (78.6) \| 311 (69.6) \| CMS Hospital General Information File \| \| **Utilization characteristics** \|  \|  \|  \| \| **ED visits** \| 3 (7.1) \| 7 (1.6) \| AHA (VEM) \| \| **Observation days** \| 0 (0.0) \| 0 (0.0) \| Cost Reports (NMRC, S300001, 2800, 800) \| \| **Inpatient days** \| 0 (0.0) \| 0 (0.0) \| Cost Reports (NMRC, S300001, 100, 800) \| \| **ICU days** \| 0 (0.0) \| 0 (0.0) \| Cost Reports (NMRC, S300001, 800, 800) \| \| **Case mix index** \| 21 (50.0) \| 338 (75.6) \| WRDS (CMI) \| \| **Staffing characteristics** \|  \|  \|  \| \| **Physician privileges** \| 18 (42.9) \| 184 (41.2) \| AHA (TPRTOT) \| \| **Hospitalists provide care** \| 17 (40.5) \| 165 (36.9) \| AHA (HSPTL) \| \| **Full time RN** \| 3 (7.1) \| 7 (1.6) \| AHA (FTRNTF) \| \| **Full time RT** \| 3 (7.1) \| 7 (1.6) \| AHA (FTRESP) \| \| **Full time Pharmacist** \| 3 (7.1) \| 7 (1.6) \| AHA (FTPHR) \| \| **Service availability** \|  \|  \|  \| \| **Neurological Services** \| 15 (35.7) \| 138 (30.9) \| AHA (NEROHOS, NEROSYS, NEROVEN) \| \| **Oncology Services** \| 15 (35.7) \| 138 (30.9) \| AHA (ONCOLHOS, ONCOLSYS, ONCOVEN) \| \| **Orthopedic Services** \| 15 (35.7) \| 138 (30.9) \| AHA (ORTOHOS, ORTOSYS, ORTOVEN) \| \| **Cardiology Services** \| 15 (35.7) \| 138 (30.9) \| AHA (ACARDHOS, ACARDSYS, ACARDVEN) \| \| **Outpatient Surgery** \| 15 (35.7) \| 138 (30.9) \| AHA (OPSRGHOS, OPSRGSYS, OPSRGVEN) \| \| **Hemodialysis** \| 15 (35.7) \| 138 (30.9) \| AHA (HEMOHOS, HEMOSYS, HEMOVEN) \| \| **Financial characteristics** \|  \|  \|  \| \| **Total margin** \| 1 (2.4) \| 9 (2.0) \| WRDS (EXMAR) \| \| **Operating margin** \| 1 (2.4) \| 9 (2.0) \| WRDS (OPMAR) \| \| **Days cash on hand** \| 0 (0.0) \| 1 (0.2) \| WRDS (DCOH) \| \| **Current ratio** \| 1 (2.4) \| 12 (2.7) \| WRDS (CURRAT) \| \| **Uncompensated care** \| 0 (0.0) \| 1 (0.2) \| WRDS (UNCOMCARCOS / OPEREX) \| \| Abbreviations used: AHA, American Hospital Association; CMS, Centers for Medicare and Medicaid Services; ED, Emergency Department; ICU, intensive care unit; REH, Rural Emergency Hospital; RN, registered nurse; RT, respiratory therapist; WRDS, Wharton Research Data Services  ^a^Codes for Medicare Cost Reports are reported as form, sheet, line, and column.  ^b^In the AHA Survey, a blank (i.e., NA) response to health system membership does not distinguish between non-membership or missing. \| \| \| \| |
| --- | --- | --- | --- | --- | --- | --- | --- | --- | --- | --- | --- | --- | --- | --- | --- | --- | --- | --- | --- | --- | --- | --- | --- | --- | --- | --- | --- | --- | --- | --- | --- | --- | --- | --- | --- | --- | --- | --- | --- | --- | --- | --- | --- | --- | --- | --- | --- | --- | --- | --- | --- | --- | --- | --- | --- | --- | --- | --- | --- | --- | --- | --- | --- | --- | --- | --- | --- | --- | --- | --- | --- | --- | --- | --- | --- | --- | --- | --- | --- | --- | --- | --- | --- | --- | --- | --- | --- | --- | --- | --- | --- | --- | --- | --- | --- | --- | --- | --- | --- | --- | --- | --- | --- | --- | --- | --- | --- | --- | --- | --- | --- | --- | --- | --- | --- | --- | --- | --- | --- | --- | --- | --- | --- | --- | --- | --- | --- | --- | --- | --- | --- | --- | --- | --- | --- | --- | --- | --- | --- | --- | --- |
|  |

**eReferences**

1. Cost Reports. Centers for Medicare and Medicaid Services. Accessed April 17, 2025. <https://www.cms.gov/data-research/statistics-trends-and-reports/cost-reports>

2. Grambrel R. Extracting Variables from Cost Reports. The Comprehensive R Archive Network. Accessed April 17, 2025. <https://cran.r-project.org/web/packages/medicare/vignettes/cost-report-analysis.html>

3. Wharton Research Data Services. The Wharton School of the University of Pennsylvania. Accessed January 2, 2026. [wrds.wharton.upenn.edu](http://wrds.wharton.upenn.edu/)

4. Hospitals archived data snapshots. Centers for Medicare and Medicaid Services. Accessed February 18, 2026. <https://data.cms.gov/provider-data/archived-data/hospitals>

5. Hospital Enrollments. Centers for Medicare and Medicaid Services. Accessed January 2, 2026. <https://data.cms.gov/provider-characteristics/hospitals-and-other-facilities/hospital-enrollments>
